# The bidirectional association between the disability in activities of daily living and depression: a longitudinal study in Chinese middle-aged and older adults

**DOI:** 10.1186/s12889-024-19421-w

**Published:** 2024-07-15

**Authors:** Lina Zhou, Wei Wang, Xiancang Ma

**Affiliations:** Department of Psychiatry, The First Affiliated Hospital of Xi’an Jiaotong University, 277 West Yanta Road, Xi’an, Shaanxi P. R. China

**Keywords:** Activities of daily living, Chinese, Depression, Middle-aged; older adults

## Abstract

**Aim:**

Depression and disability in activities of daily living (ADL) are common in middle-aged and older adults. This study investigated the bidirectional relationship between depression and disability in ADL in Chinese middle-aged and older adults.

**Methods:**

Data from a baseline study of 17,596 participants from the China Health and Retirement Longitudinal Study (CHARLS) and two follow-up visits at 4 and 7 years were included. We designed Study A and Study B to explore the interaction between depression and disability in ADL in middle-aged and older people.

**Results:**

Individuals with disability in ADL at baseline had adjusted odds ratios (ORs) of 1.331 (1.118, 1.584) and 1.969 (1.585, 2.448) for developing depression compared with those without disability in ADL at the 4- and 7-year follow-ups, respectively. Individuals with depression at baseline had adjusted ORs of 1.353 (1.127, 1.625) and 1.347 (1.130, 1.604), respectively, for developing disability in ADL 4 and 7 years later.

**Conclusions:**

There was a bidirectional relationship between depression and disability in ADL. Depression increased the risk of disability in ADL, but this risk did not increase with time, whereas the effect of disability in ADL on depression increased with time.

## Introduction

Depression is the mental illness with the highest incidence globally. The lifetime prevalence of depression is 3.4%, and the incidence in middle-aged and older people over the age of 50 years is significantly higher than that in adolescents and young adults in mainland China [1, 2]. China is the most populous country in the world, and it has a growing number of older people [3, 4]. Therefore, the Chinese government will need to confront the issues of mental health and mental illness, particularly depression, in the near future.

Depression is reportedly associated with functional impairment [5]. Carrière et al. [6] demonstrated that depression is an independent predictor of disability in the older population. Furthermore, a prospective cohort study in Japan indicated that depressive symptoms are associated with future dependence in activities of daily living (ADL) in older adults, over a 7.5-year study period [7]. Disability in ADL caused by depression is also affected by several factors related to ADL. Additionally, older age is usually accompanied by various physical diseases or symptoms, which may also lead to a disability in ADL, and the combination of depression with such diseases often aggravates an existing disability in ADL [8–10]. Thus, improvements in depression in older individuals may also help to improve their daily functioning. For example, a study found a correlation between an improvement in depressive symptoms and a reduction in falls [11]. On the other hand, disability in ADL may play a significant role in the occurrence of depression among older people. Some studies have found that disability in ADL has the potential to increase the risk of depressive symptoms in middle-aged and older Chinese adults and their spouses [12, 13]. Moreover, there might be a threshold in the severity of disability that triggers the development of depressive symptoms [14]. In contrast, the levels of social support and abilities in ADL among older individuals with a high quality of life were higher, and their levels of depression were lower [15]. Higher functional status and physical activity play potentially important positive roles in the prevention of depression among older individuals [16, 17]. It is worth noting that disability and less support with activities, which is associated with disability in ADL [18, 19], can also contribute to a greater recurrence risk of late-life depression [20, 21].

There seems to be a bidirectional relationship between disability in ADL and depression in middle-aged and older people [22]. However, most previous studies have been cross-sectional, making it difficult to determine cause and effect. In addition, depression and ADL in older individuals are affected by many factors, such as gender, age, marital status, chronic diseases, physical disability, cognitive impairment, and even regional and ethnic factors [5, 23–26].Therefore, in this study, we implemented a large national cohort study, the China Health and Retirement Longitudinal Study (CHARLS), which conducted a baseline survey and two follow-up visits on the Chinese mainland, to explore the relationship between disability in ADL and depression in middle-aged and older people.

## Methods

### Study design

The CHARLS is a community study conducted in mainland China in individuals aged ≥ 45 years. Currently, researchers have conducted a baseline survey in 2011 and three follow-up visits in 2013, 2015, and 2018, mainly covering economic, social, health, and other aspects [27]. The CHARLS database is freely available worldwide, and the authors of this study applied online for authorization for use in scientific analysis(https://charls.pku.edu.cn/).

We analyzed the bidirectional association of disability in ADL and depression from two aspects, namely Study A and Study B, based on the large community cohort of CHARLS. The purpose of Study A was to explore whether disability in ADL can lead to depression. Therefore, we selected individuals without depression at baseline and divided them into those with and without disability in ADL at baseline and compared their depression levels at two follow-up visits in 2015 and 2018. The purpose of Study B was to explore the impact of depression on ADL. In individuals with and without depression and with normal ADL at baseline, we compared their ADL in the 2015 and 2018 follow-up visits.

### Study population

#### Study A: association of baseline disability in ADL with follow‑up depression

**Fig. 1:**
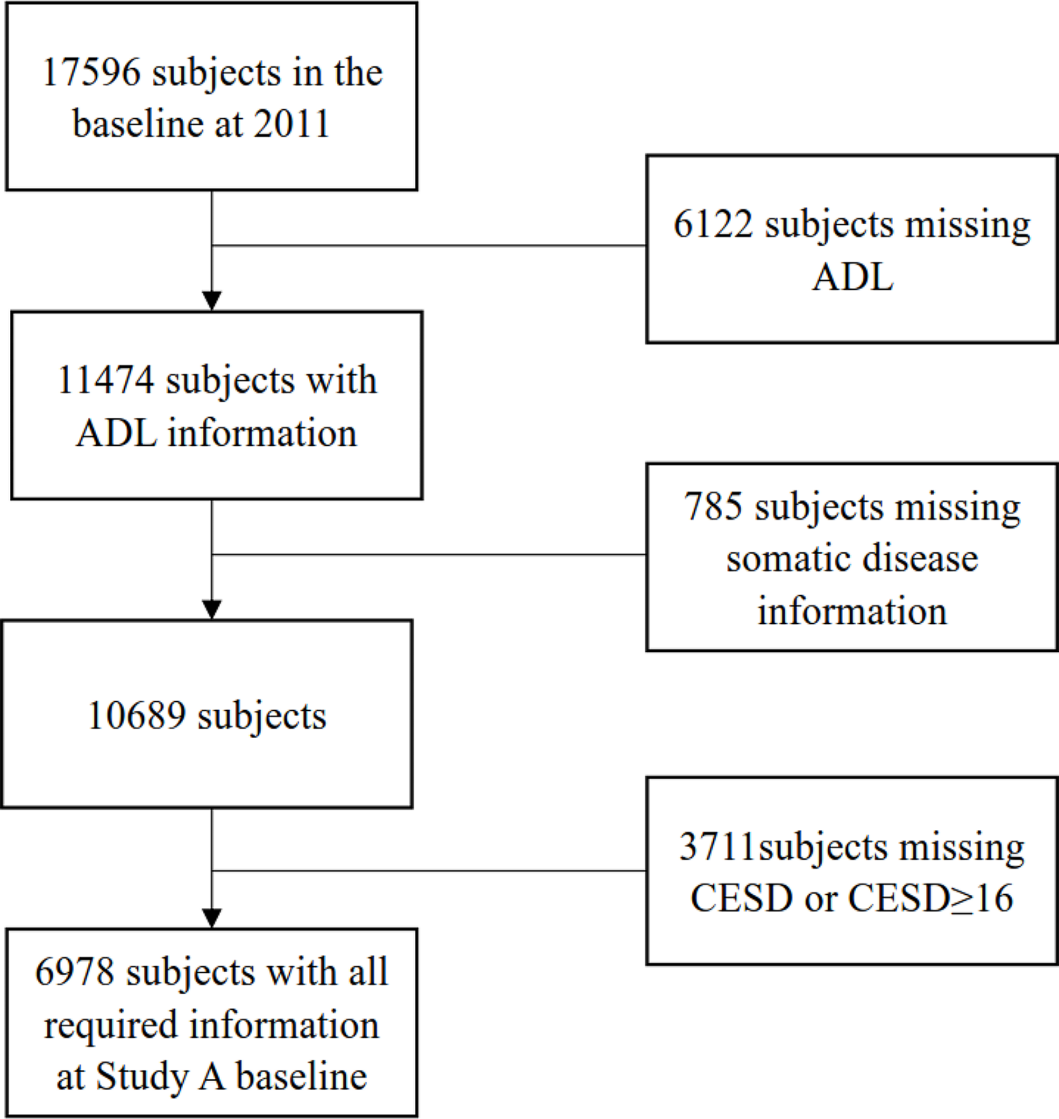
Flow chart of the selection of the study population for Study A ADL, activities of daily living; CESD, Center for Epidemiological Survey Depression Scale

In Study A, those subjects who met the following criteria were selected: (1) no depression at baseline, by Center for Epidemiological Survey Depression Scale (CESD) score < 16, (2) complete baseline demographic and chronic disease data, CESD score, and ADL score, and (3) CESD scores in 2015 and 2018 were required for patients who had completed the follow-ups. Consequently, 6,978 participants were included in the subsequent analysis (Fig. 1).

#### Study B: association of baseline depression with follow‑up disability in ADL

**Fig. 2:**
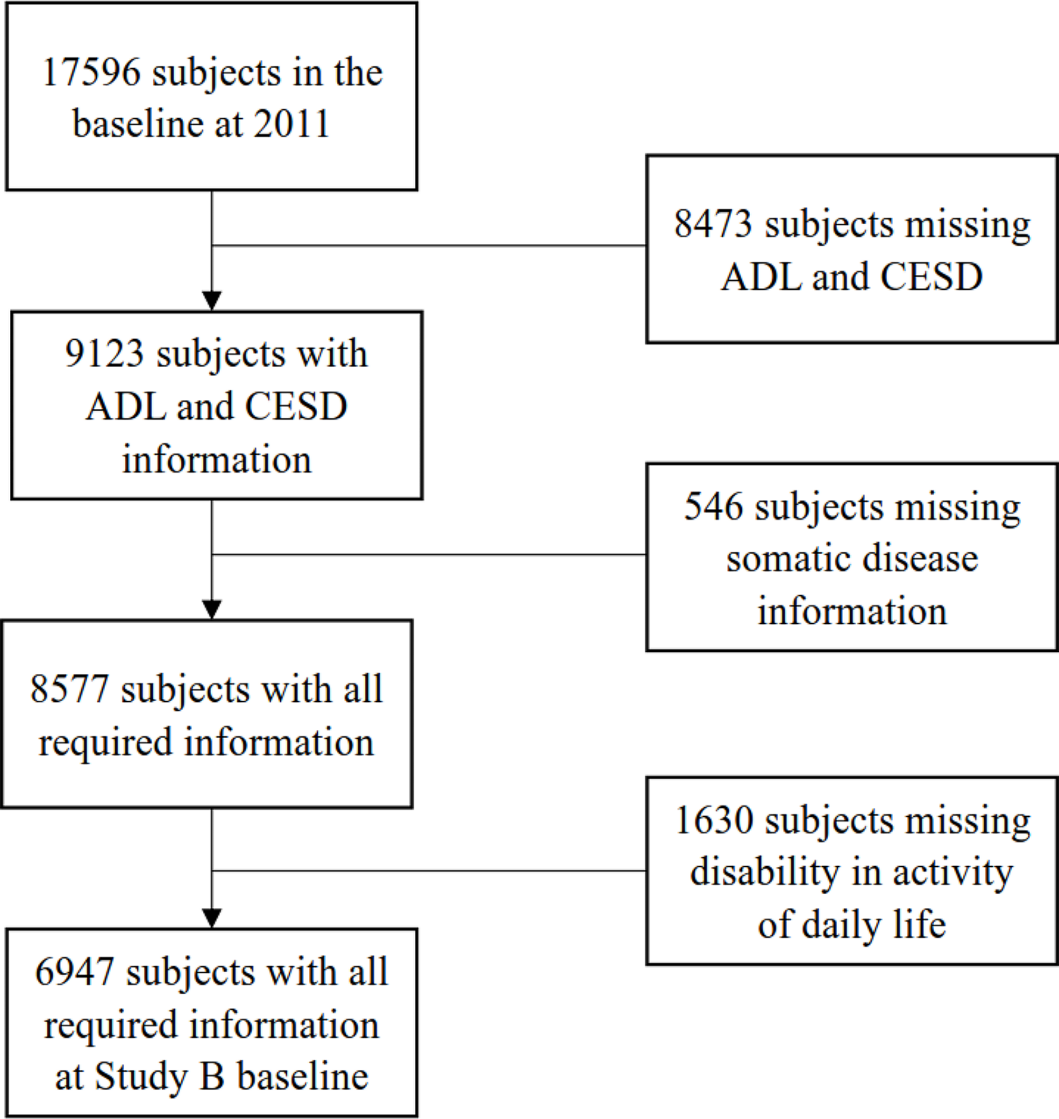
Flow chart of the selection of the study population for Study B ADL, activities of daily living; CESD, Center for Epidemiological Survey Depression Scale

In Study B, the inclusion criteria were as follows: (1) no disability in ADL at baseline, (2) complete baseline demographic and chronic disease information, CESD score, and ADL score, and (3) ADL scores in 2015 and 2018 were required for patients who had completed follow-ups. Finally, 6,947 participants were included in the subsequent analysis (Fig. 2).

### Activities of daily living (ADL)

The Physical Self-Maintenance Scale (PSMS) and Instrumental Activities of Daily Living Scale (IADL) were used to assess an individual’s ADL. The PSMS consists of six items on the performance of the physical activities including using the toilet, self-feeding, self-dressing, self-grooming, self-ambulating, and self-bathing. The IADL covers five items, including shopping, preparing food, housekeeping, taking medicine, and managing finances. According to the degree of disability, the scores represent difficulty (score 1), difficulty but the activity can be completed independently (score 2), help needed to complete the activity (score 3), and completely unable to complete the activity (score 4). If any one of the 11 items had a score ≥ 3, the person was considered to have a disability in ADL; otherwise, the person was considered as having no disability in ADL [28].

### Depressive symptoms

The 10-item CESD, which is widely used in large epidemiological investigations, was used to assess depressive symptoms. The respondents were asked to rate “how often you felt this way during the past week,” with scores ranging from 0 to 30. According to previous reports [29], a cutoff of 16 was used to identify individuals with depression. In other words, a CESD score ≥ 16 was defined as depression, and a CESD score < 16 was defined as no depression. The Chinese version of the CESD has been applied to older populations in China and has good reliability and validity [30].

### Statistical analysis

All data in this study were obtained from the CHARLS database. The data in DTA format were converted into XLS format by STATA MP 16.0 (StataCorp LLC, College Station, TX, US) and then imported into SPSS 26.0 (SPSS Inc., Chicago, IL, USA) for analysis.

All baseline data were compared between groups, including baseline with or without disability in ADL and baseline data with or without depression. Categorical variables are presented as N, and the chi-square test was used for comparisons between groups. Continuous variables are presented as mean ± standard deviation (M ± SD), and t-tests were used for comparison between groups.

To investigate the impact of disability in ADL at baseline on subsequent depression, and the impact of depression at baseline on subsequent disability in ADL, we conducted multiple logistic regression and multiple linear regression analyses, respectively. The results are presented as odds ratios (OR) and 95% confidence intervals (95%CIs). In all results, *P* < 0.05 was used as the criterion for statistically significant differences.

## Results

### Study A: association of baseline disability in ADL with follow‑up depression

#### Baseline information in 2011

In Study A, 5,910 (84.69%) subjects had no disability in ADL (No-disability group) and 1,068 (15.31%) subjects had disability in ADL (Disability group). The baseline information of these subjects showed significant differences in age, education, marital status, smoking, drinking, combined with physical disability, visual and hearing impairment, sleep disorders, and chronic diseases (all *P* < 0.05). The CESD scores were higher in subjects with disability in ADL than in those without (*P* < 0.001), although these scores were not at a diagnostic level of depression. The detailed data are shown in Table 1.

**Table 1:** Difference of Study A baseline data between participants with and without disability in ADL

		No disability in ADL (*n* = 5910)	Disability in ADL (*n* = 1068)	F/t	*P* value
Age, years, M ± SD		62.68 ± 76.73	72.04 ± 119.39	-2.473	**0.014**
Gender, n					
	Male	2578	458	0.200	0.655
	Female	3332	610		
Education, n					
	0 years	1552	471	155.72	**< 0.001**
	1–6 years	2500	399		
	7–12 years	1732	185		
	> 12 years	126	13		
Marital status, n					
	Married	5175	876	6978.00	**< 0.001**
	Separated or divorced	59	10		
	Widowed or never married	676	182		
Physical disability, n					
	No	5721	952	127.092	**< 0.001**
	Yes	189	116		
Visual impairment, n					
	No	5610	939	76.871	**< 0.001**
	Yes	300	129		
Hearing impairment, n					
	No	5566	904	121.837	**< 0.001**
	Yes	344	164		
Sleep disorders, n					
	No	5908	1058	42.915	**< 0.001**
	Yes	2	2		
Chronic diseases, M ± SD		1.44 ± 1.34	2.00 ± 1.58	-10.875	**< 0.001**
Smoking, n					
	No	2172	399	0.144	0.705
	Yes	3738	669		
Drinking, n					
	No	1324	180	16.471	**< 0.001**
	Yes	4586	888		
CESD score, M ± SD		7.31 ± 4.08	9.25 ± 3.96	-14.394	**< 0.001**
PSMS score, M ± SD		6.18 ± 0.59	8.29 ± 3.10	-22.290	**< 0.001**
IADL score, M ± SD		5.17 ± 0.58	9.88 ± 3.60	-42.551	**< 0.001**
ADL score, M ± SD		11.31 ± 1.14	18.08 ± 5.81	-37.946	**< 0.001**

ADL, activities of daily living; CESD, Center for Epidemiological Survey Depression Scale; IADL, instrumental activities of daily living; M, mean; PSMS, Physical Self-Maintenance Scale; SD, standard deviation.

#### Four-year follow-up in 2015

In the follow-up conducted in 2015, 5,053 (72.41%) subjects completed the follow-up, including 4,405 (87.18%) subjects without disability in ADL and 648 (12.82%) subjects with disability in ADL. The average CESD score of the latter was significantly higher than that of the former in 2015 (17.98 ± 6.60 vs. 16.02 ± 6.00, t = -7.645, *P* < 0.001). However, there was no difference between the two groups in the average increase in CESD score after 4 years (8.71 ± 6.07 vs. 8.83 ± 6.39, t = -0.431, *P* = 0.666).

Of the 4,405 subjects without disability in ADL at baseline, 1,982 (44.99%) reached the diagnostic level of depression (CESD score ≥ 16), and 2,423 (55.01%) did not. Of the 648 (12.82%) individuals with disability in ADL, 366 (56.48%) met the diagnostic criteria for depression, and 282 (43.52%) did not. Subjects with disability in ADL at baseline showed an increased prevalence of depression at 4 years of follow-up (F = 29.966, *P* < 0.001). Logistic regression analysis showed that individuals with disability in ADL at baseline had an adjusted OR (95% CI) of 1.331 (1.118,1.584) for developing depression compared with those without disability (Table 2).

To explore which factors at baseline might influence subsequent depression, we performed multiple linear regression analyses that showed CESD score at baseline, gender, number of chronic diseases, PSMS score at baseline, and smoking were positively correlated with CESD score at the 2015 follow-up. Education was negatively correlated with CESD score in 2015. Independent of other factors, PSMS score were significantly associated with an increase in CESD score 4 years later (0.194 [0.073‒ 0.315]).

#### Seven-year follow-up in 2018

In the 2018 follow-up, 4,323 (61.95%) individuals at baseline were followed up, of whom 3,821 (88.39%) had no disability in ADL at baseline and 502 (11.61%) had a disability in ADL. The CESD scores of subjects with disability in ADL at the 2018 follow-up were significantly higher than those of subjects without such disability (10.87 ± 6.82 vs. 8.77 ± 6.29, t = -6.947, *P* < 0.001). There was no significant difference in average CESD score increase after 7 years between the two groups (1.45 ± 6.40 vs. 1.71 ± 6.81, t = -0.789, *P* = 0.432).

Of those without disabilities, 586 (15.34%) had a diagnosis of depression at the follow-up 7 years later, whereas 3,235 (84.66%) did not. Of the patients with disability in ADL, 132 (26.29%) met the diagnostic level of depression after 7 years, whereas 370 (73.71%) did not. Individuals with disability in ADL also showed increased rates of depression after 7 years (F = 38.472, *P* < 0.001). Individuals with disability in ADL at baseline had an adjusted OR (95% CI) of 1.969 (1.585, 2.448) for developing depression after 7 years compared with those without disability in ADL (Table 2).

**Table 2:** Adjusted odds ratios of depression for disability in ADL and disability in ADL for depression

		2015		2018	
		OR(95%CI)	*P* value	OR(95%CI)	*P* value
Study A					
	No disability in ADL	1.000	-	1.000	-
	Disability in ADL	1.331(1.118,1.584)	**0.001**	1.969 (1.585, 2.448)	**< 0.001**
Study B					
	No-depression	1.000	-	1.000	-
	Depression	1.353(1.127,1.625)	**0.001**	1.347(1.130,1.604)	**0.001**

ADL, activities of daily living; CI, confidence interval; OR, odds ratio.

Moreover, we found that CESD score at baseline, gender, education, number of chronic diseases, IADL score at baseline, and visual impairment were correlated with CESD score at the 2018 follow-up. In contrast to the 2015 follow-up, we found that the IADL score at baseline, rather than the PSMS score, was associated with an increase in the CESD score by 2018, 7 years later (OR[95%CI], 0.205 [0.090, 0.320]).

### Study B: association of baseline depression with follow‑up disability in ADL

#### Baseline information in 2011

In Study B, 5,910 (85.07%) subjects with depression and 1,037(14.93%) subjects without depression at baseline were included. The two groups differed in terms of gender, education, marital status, physical disability, visual and hearing impairment, sleep disorders, and chronic diseases (*P* < 0.05). The PSMS, IADL, and ADL scores were higher in subjects with depression than in subjects without depression (*P* < 0.001). The detailed data are shown in Table 3.

**Table 3:** Difference of Study B baseline data between participants with and without depression

		No-depression (*n* = 5910)	Depression (*n* = 1037)	F/t	*P* value
Age, years, M ± SD		62.68 ± 76.73	63.77 ± 86.17	-0.417	0.677
Gender, n					
	Male	2578	384	15.670	**< 0.001**
	Female	3332	653		
Education, n					
	0 years	1552	377	90.854	**< 0.001**
	16 years	2500	471		
	712 years	1731	185		
	> 12 years	127	4		
Marital status, n					
	Married	5175	821	55.349	**< 0.001**
	Separated or divorced	59	24		
	Widowed or never married	676	192		
Physical disability, n					
	No	5721	975	19.589	**< 0.001**
	Yes	189	62		
Visual impairment, n					
	No	5610	932	40.968	**< 0.001**
	Yes	300	105		
Hearing impairment, n					
	No	5566	918	45.349	**< 0.001**
	Yes	344	119		
Sleep disorders, n					
	No	5908	1035	3.877	**0.049**
	Yes	2	2		
Chronic diseases, M ± SD		1.44 ± 1.34	1.92 ± 1.55	-9.354	**< 0.001**
Smoking, n					
	No	3738	677	1.578	0.209
	Yes	2172	360		
Drinking, n					
	No	4586	828	2.593	0.107
	Yes	1324	209		
CESD score, M ± SD		7.31 ± 4.08	19.11 ± 2.90	-113.058	**< 0.001**
PSMS, M ± SD		6.18 ± 0.59	6.55 ± 1.08	-10.880	**< 0.001**
IADL, M ± SD		5.17 ± 0.58	5.47 ± 0.99	-9.597	**< 0.001**
ADL, M ± SD		11.34 ± 0.98	12.02 ± 1.83	-11.634	**< 0.001**

ADL, activities of daily living; CESD, Center for Epidemiological Survey Depression Scale; IADL, instrumental activities of daily living; M, mean; PSMS, Physical Self-Maintenance Scale; SD, standard deviation.

#### Four-year follow-up in 2015

During the 2015 follow-up, 4,719 (67.93%) individuals completed the follow-up, including 3,937 (83.43%) without depression at baseline and 782 (16.57%) with depression at baseline. In the four-year follow-up, PSMS (6.68 ± 2.10 vs. 7.15 ± 2.04), IADL (6.01 ± 2.36 vs. 6.65 ± 2.74), and ADL (12.70 ± 3.73 vs. 13.80 ± 4.29) scores of individuals with depression at baseline were significantly higher than those of individuals without depression at baseline (all *P* < 0.001). In addition, the average increases in IADL (0.83 ± 2.36 vs. 1.15 ± 2.76, t = -3.041, *P* = 0.002) and ADL (1.32 ± 3.73 vs. 1.74 ± 4.28, t = -2.574, *P* = 0.010) scores of participants with depression at baseline were higher than those of participants without depression at baseline, whereas the mean increase in PSMS scores showed no difference after 4 years (0.49 ± 1.76 vs. 0.59 ± 2.07, t = -1.288, *P* = 0.198).

Of the individuals without depression at baseline, 751 (19.08%) showed disability in ADL during the follow-up after 4 years, and 3,186 (80.92%) had no disability. Among the individuals with depression at baseline, 223 (28.52%) developed a disability in ADL by the 4-year follow-up, and 559 (71.48%) did not. The incidence of disability in ADL was significantly higher in individuals with than in those without depression at baseline (F = 35.503, *P* < 0.001). Logistic regression analysis showed that individuals with depression at baseline had an adjusted OR (95%CI) of 1.353 (1.127,1.625) that they would develop disability in ADL 4 years later (Table 2).

Furthermore, ADL score at baseline, education, CESD score at baseline, number of chronic diseases, hearing and visual impairment, and marital status showed correlations with ADL score. Among them, the CESD score at baseline was positively related to the ADL score at the 2015 follow-up, independent of other factors (OR[95%CI], 0.038 [0.019, 0.058]).

#### Seven-year follow-up in 2018

A total of 4,584 (65.99%) participants completed the follow-up in 2018, including 3,848 (83.94%) without and 736 (16.06%) with depression at baseline. PSMS (6.83 ± 2.10 vs. 7.38 ± 2.27), IADL (6.46 ± 3.00 vs. 7.13 ± 3.27), and ADL (13.29 ± 4.68 vs. 14.51 ± 5.02) scores were significantly higher in individuals with than in individuals without depression at baseline (*P* < 0.001). After 7 years, the mean increases in PSMS (0.64 ± 2.12 vs. 0.85 ± 2.35, t = -2.278, *P* = 0.023), IADL (1.29 ± 3.01 vs. 1.68 ± 3.29, t = -2.998, *P* = 0.003), and ADL (1.93 ± 4.68 vs. 2.53 ± 5.06, t = -2.996, *P* = 0.003) scores in the group with depression at baseline were all higher than those in the group without depression at baseline.

Of the individuals without depression at baseline, 930 (24.17%) had a disability in ADL by the 7-year follow-up, and 2,918 (63.66%) had no disability in ADL. Of the individuals with depression at baseline, 258 (35.05%) had developed a disability in ADL by the 7-year follow-up, and 478 (64.95%) had not. The incidence of disability in ADL was significantly higher in individuals with than in those without depression at baseline (F = 38.133, *P* < 0.001). Individuals with depression at baseline had an adjusted OR of 1.347 (1.130, 1.604) of developing disability in ADL after 7 years (Table 2).

Moreover, ADL score at baseline, education, number of chronic diseases, marital status, CESD score at baseline, and gender showed a significant relationship with ADL score at the 2018 follow-up. Also, the CESD score was independent of the other factors (0.050 [0.026, 0.074]).

## Discussion

A large number of previous cross-sectional studies have found that disability in ADL and depression affect each other in middle-aged and older adults [6, 7, 16, 20, 23]. To explore this issue, we assessed the ADL and depression levels at baseline and at 4- and 7-year follow-up in a large cohort study. We also took into account many demographic and physical health factors that were reported in previous studies to have an effect on depression and ADL and assessed both the impact of disability in ADL on depression and the effects of depression on disability in ADL. In general, this study demonstrated the bidirectional association between depression and disability in ADL, independent of demographics, lifestyle, physical disease, function, and other factors.

In terms of the role of disability in ADL in the subsequent depression development of depression, our results showed that the group with disability in ADL at baseline had higher depression scores than those without such disabilities, and there were also differences in their accompanying demographic and physical functioning, such as higher age, lower education level, a differing marital status, the presence of combined physical disability, impaired function, and a higher number of chronic diseases. At both the 4- and 7-year follow-ups, depression scores remained significantly higher in the disability group than in the no-disability group, and the prevalence of depression was significantly higher in the disability group than in the no-disability group. However, there was no difference in the increase in scores between the two groups, suggesting that the higher prevalence of depression in the disability group was mainly related to baseline demographic factors, physical health, and depression levels. Therefore, we performed a linear regression analysis, excluding the effects of baseline depression scores and other factors, and found an increased correlation between baseline PSMS score and CESD score at the 4-year follow-up, independent of other factors. At the 7-year follow-up, we found a stronger correlation between CESD score and baseline IADL. This indicated that the long-term depression level was more significantly affected by the IADL score. In addition, we conducted multiple logistic regression analysis to determine the impact of disability in ADL at baseline on the risk of subsequent depression. We found that the impact of disability in ADL on the risk of depression significantly increased over time, with an OR of 1.969 at the follow-up at 7 years compared to 1.331 at the follow-up at 4 years. Taken together, the ability to perform daily tasks was associated with an increased risk of developing depression, and the effect was more pronounced over time.

There are various mechanisms by which depression can be caused by impaired ADL as follows. (1) Disability in ADL leads directly to reduced social activities and social participation. Consequently, perceived social isolation and health outcomes, including cardiovascular disease and depression, worsen in older adults [31–33]. (2) People need to turn to others for help due to disability in ADL, which can lead to feelings of shame and increased guilt and self-blame [34, 35]. (3) People are often unable to perform work or perform the tasks that are necessary for ADL; thus, they become financially burdened, increasing their risk of depression [36]. (4) The reduced quality of life prevents them from feeling happy and fulfilled [37, 38]. (5) Their chronic disease management may be worse, and the aggravation of chronic disease may also lead to depression [10]. (6) Caregiver burden had a 40.0% indirect effect on caregivers’ life satisfaction, due to caregiver depression [13], which also indirectly affects the mood of the person receiving care [39]. The disability in ADL not only leads to individual depression but is also considered to be an independent risk factor for suicidal ideation [40]. In addition, other studies have suggested that older individuals with normal ADL are at an increased risk of impaired ADL if they experience reduced social interaction [41] and increased loneliness [38]. This may further increase the risk of depression, in conjunction with many of the reasons mentioned above.

Furthermore, we explored the impact of depression on subsequent ADL. The results showed that those with depression had higher baseline PSMS, IADL, and ADL scores than those without depression, even though they did not meet the criteria for daily dysfunction. Similarly, people with depression differed from those without depression in terms of social demography, physical health, and other factors. For instance, they were more often females, had lower education levels, a differing marital status, more physical disability, impaired function, and a higher number of chronic diseases. These factors have previously been reported to be associated with the development of depression [42, 43] and disability in ADL [44]. During the follow-up after 4 years, we found that, in addition to higher PSMS, IADL, and ADL scores, and more subjects with disability in ADL, the average increase in IADL and ADL scores of the depression group were also significantly higher than those of the no-depression group. After excluding the influence of ADL score, sociodemographic factors, physical health, and other factors at baseline, we found that the association between ADL and CESD scores did not increase. This suggests that the ADL of patients with depression may be gradually aggravated by the deterioration of physical health status as they grow older. After 7 years of follow-up, the PSMS, IADL, and ADL scores in the depression group were all higher than those in the no-depression group. The prevalence of disability in ADL in the depression group was also higher than that in the no-depression group, and the correlation between ADL and CESD scores was increased. This suggests that the correlation between ADL and CESD scores is more significant over time. Furthermore, we found that individuals with depression at baseline had a similar risk of impaired ADL after 4 and 7 years, with OR values of 1.353 and 1.347, respectively, indicating that the impact of depression on subsequent ADL was stable and relatively lasting.

Reasons for the disability in ADL caused by depression may include, first, the characteristics of depression. Patients with depression have decreased interest and motivation, leading to a decrease in their participation in sports and social activities. This decline in functional exercise will directly lead to a disability in ADL [45]. Second, depression is often accompanied by cognitive impairment, which can lead to worse functioning, such as in financial management and cooking [46]. Moreover, middle-aged and older people are at high risk of cognitive impairment [47, 48], which may further lead to the deterioration of patients’ daily functioning. In addition, poor sleep may be a robust and independent risk factor for disability in adults of all ages [49], and the incidence of sleep disorders increases with age [50]. It is worth noting that our study found that the impact of depression on daily functioning became more pronounced with age. This may be related to indirect changes associated with increasing age, such as a less active state, impaired cognitive function [51], living alone, or living with non-spouse others [52].

In summary, this investigation found a bidirectional relationship between depression and disability in ADL. Both depression levels at baseline and ADL were associated with depression levels and functioning in ADL 4 and 7 years later. Therefore, in China’s aging society, it is crucial to pay attention to depression in middle-aged and older individuals and intervene as soon as possible. It should be noted that this study used data of 4 and 7 years of follow-up, and did not include more follow-up stages. Although the results showed the correlation between disability in ADL and depression, they still lacked detailed further investigation, and we hope to improve them in subsequent studies.

## Data Availability

Data for this study were obtained from a publicly available database in China, which was available on request.

## Abbreviations

ADL: Activities of daily living
CESD: Center for Epidemiological Survey Depression Scale
IADL: Instrumental activities of daily living
PSMS: Physical Self-Maintenance Scale
M: Mean
SD: Standard deviation
CI: Confidence interval
OR: Odds ratio
